# Sex differences in gut microbiota composition, function, and assembly in the plateau zokor (*Eospalax baileyi*)

**DOI:** 10.7717/peerj.20646

**Published:** 2026-01-26

**Authors:** Lei Si, Rui Zhang, Jialong Guo, Haijing Wang, Jingyan Yan, Daoxin Liu

**Affiliations:** 1 State Key Laboratory of Plateau Ecology and Agriculture, Qinghai University, Xining, Qinghai Province, China; 2 School of Ecological and Environmental Engineering, Qinghai University, Xining, Qinghai Province, China; 3 College of Agriculture and Animal Husbandry, Qinghai University, Xining, Qinghai Province, China; 4 Department of Medicine, Qinghai University, Xining, Qinghai Province, China

**Keywords:** Plateau zokor, Gut microbiota, 16S rRNA, Functional prediction, Assembly processes

## Abstract

**Background:**

Gut microbiota play a vital role in nutrient metabolism, immune regulation, and host homeostasis. However, the role of sex differences in shaping the gut microbiota of plateau zokors (*Eospalax baileyi*) remains unclear. The present study aims to explore how sex influences the composition, function, and assembly processes of the gut microbiota in plateau zokors.

**Methods:**

In this study, we performed Illumina 16S rRNA (V3–V4) sequencing on 15 gastrointestinal samples to assess sex-related differences in gut bacterial diversity, function, and community assembly.

**Results:**

No significant differences were observed in species richness or diversity between males and females; however, the gut microbial community structures differed significantly by sex (*p* < 0.01). At the phylum level, both sexes shared dominant phyla, including Firmicutes, Desulfobacterota, and Bacteroidota. Across both the phylum and genus levels, males and females shared the same dominant taxa, yet their relative abundances exhibited clear sex-specific differences. PICRUSt-based functional prediction indicated that the gut microbiota were mainly associated with energy metabolism, DNA repair, and cellular defense. Significant sex-related differences were detected in metabolic functions (*p* < 0.05), with males showing higher carbohydrate metabolism (*p* < 0.05), while females exhibited stronger xenobiotic biodegradation and metabolism (*p* < 0.05). Neutral community model (NCM) analysis showed that males (Nm = 228.21) had higher Nm values than females (Nm = 213.44), indicating greater microbial dispersal among males. Standardized neutrality score (NST) values (<0.5) indicated that deterministic processes predominantly governed community assembly in both sexes, with males exhibiting significantly lower values than females (*p* < 0.001). iCAMP analysis further revealed that drift and dispersal limitation were the primary assembly processes, with significant sex-related differences (*p* < 0.001).

**Conclusion:**

Sex differences markedly influence gut microbial structure, functions, and assembly processes in plateau zokors, offering new insights into the adaptive evolution of this species in cold, hypoxic environments.

## Introduction

The gut microbiota are composed primarily of bacteria, along with fungi, archaea, viruses, and eukaryotic organisms, among which bacteria represent the dominant fraction (Derrien, Alvarez & Vos, 2019). Gut microorganisms play essential roles in host nutrient metabolism, immune regulation, and the maintenance of homeostasis. At the same time, their community structure is shaped by both host-intrinsic and environmental factors, including diet, host genotype, and geographical distance (Raymann & Moran, 2018; Ewen, 2017; Tremaroli & Bäckhed, 2012; Li et al., 2015; Shi et al., 2023; Ding et al., 2017). In earlier studies, the influence of sex on gut microbiota was often overlooked or masked by other factors such as host genetics (Kovacs et al., 2011). However, accumulating evidence suggests that sex is one of the key factors contributing to inter-individual variation in gut microbial communities (Mendelsohn & Karas, 2005). Sex differences regulate gut microbiota composition through hormone–microbiota interactions and sex-specific immune responses (Bolnick et al., 2014). For example, Tetel et al. (2018) demonstrated that steroid hormones can directly alter gut microbial composition. Org et al. (2016) reported significant sex-specific differences in the gut microbiota of mice, with Actinobacteria and Tenericutes being more enriched in males. Similarly, Yang et al. (2018) found that both sex and seasonal variation significantly affected the gut microbial structure of the Gansu zokor. Zhu et al. (2021) observed notable sex- and age-dependent differences in the gut microbiota of giant pandas, where juvenile and adult females exhibited higher gut microbial diversity than males, whereas the trend was reversed in older individuals. In addition, Liu et al. (2024) revealed that sex influenced the assembly mechanisms of gut bacterial communities in frogs. Collectively, these findings highlight that exploring the relationship between sex, gut microbial diversity, and community assembly is essential for advancing our understanding of host adaptive evolution to environmental challenges. These findings highlight the importance of incorporating sex as a biological variable in microbiome research.

The plateau zokor (*Eospalax baileyi*) is an endemic subterranean rodent species of the Qinghai–Tibet Plateau. This species primarily inhabits alpine meadows, alpine shrubs, and alpine steppes at elevations ranging from 2,800 to 4,200 m on the plateau and surrounding high-altitude regions (Norris et al., 2004; Ma et al., 2019a; Ma et al., 2019b Chu et al., 2023). Due to its long-term residence in enclosed, dark, humid, hypoxic, and carbon dioxide-rich burrow systems, the plateau zokor exhibits degenerated vision but well-developed forelimbs, olfaction, and hearing (Nevo, 2007; Wei, 2018). As a root-eating rodent, it primarily relies on digging for foraging, with activities concentrated within 3–20 cm of the topsoil layer (Zhang & Liu, 2002). To date, studies on the gut microbiota of the plateau zokor have mainly focused on the effects of diet, seasonal variation, age, and habitat (La et al., 2025; Tan, 2024; Ren et al., 2025). However, little is known about sex-related differences in gut microbial community structure and their ecological implications.

In this study, we conducted a preliminary investigation of the gut microbiota in male and female plateau zokors using Illumina high-throughput sequencing. Specifically, we analyzed microbial diversity, predicted functional profiles, and community assembly processes to evaluate the effects of sex on gut microbial diversity, composition, and assembly mechanisms. Furthermore, we predicted the potential relationships between the microbiota and host metabolic functions. These findings provide new insights into sex-specific microbiome characteristics and establish a foundation for understanding the adaptive evolutionary strategies of plateau zokors in cold and hypoxic environments.

## Materials and Methods

### Sample collection and DNA extraction

To minimize the potential influence of genetic background, seven male and eight female plateau zokors were captured using live traps in Qinglin Township, Datong County, Qinghai Province, China (37°8′20″N, 101°15′1″E; altitude 3,111 m). All individuals were collected within a 0.5 km radius between October 1 and 3, 2020. Following capture, animals were transferred to a temporary field station and deeply anesthetized with carbon dioxide inhalation. Once the disappearance of pedal reflex confirmed the induction of anesthesia, euthanasia was performed by cervical dislocation. After death was verified, abdominal surgery was conducted under sterile conditions to collect cecal content samples, from which one 2-ml sample was obtained from each individual. which were immediately transferred into cryogenic vials, snap-frozen in liquid nitrogen, and stored for subsequent microbiota analysis.

A total of 15 samples were obtained, including female (FM; cQL001w–cQL008w) and male (M; cQL011w–cQL017w) individuals. The male group served as the control for comparison with the female group. The sample size was determined based on commonly used numbers in previous studies employing similar behavioral paradigms and rodent models, which have been sufficient to detect significant effects reliably (Xu & Zhang, 2021). All procedures were conducted postmortem to ensure that no animal experienced recovery or postoperative distress. The sampling protocol was designed to minimize animal stress and was approved by the Animal Ethics Committee of Qinghai University (Approval No. IACUC: SL-2023044). DNA from the gastrointestinal contents was extracted following the manufacturer’s instructions of the E.Z.N.A.^®^ Soil DNA Kit. As an exploratory study, no formal a priori inclusion or exclusion criteria were established. The personnel performing the sampling were aware of the group allocation and performed the group allocation during sampling. The personnel responsible for gene sequencing were blinded to the group allocation throughout the entire process. The statistician was also blinded until after the initial data analysis was completed.

### High-throughput sequencing

The V3–V4 region of the 16S rRNA gene was amplified using the universal primers 338F (5′-ACTCCTACGGGAGGCAGCAG-3′) and 806R (5′-GGACTACHVGGGTWTCTAAT-3′). Each 20 μL polymerase chain reaction (PCR) mixture contained four μL of 5 × TransStart FastPfu buffer, two μL of 2.5 mM dNTPs, 0.8 μL of forward primer (five μM), 0.8 μL of reverse primer (five μM), 0.4 μL of TransStart FastPfu DNA polymerase, 10 ng of template DNA, and nuclease-free water to a final volume of 20 μL. PCR reactions were performed in triplicate for each sample under the following conditions: initial denaturation at 95 °C for 3 min; 27 cycles of denaturation at 95 °C for 30 s, annealing at 55 °C for 30 s, and extension at 72 °C for 30 s; followed by a final extension at 72 °C for 10 min and storage at 4 °C.

### Library preparation and sequencing

Sequencing libraries were prepared using the NEXTFLEX Rapid DNA-Seq Kit according to the following steps: (1) adaptor ligation, (2) bead-based size selection to remove self-ligated adaptor fragments, (3) PCR enrichment of library templates, and (4) bead purification to obtain the final library. Paired-end sequencing (PE300) was performed on the Illumina MiSeq platform. Raw reads were processed to remove low-quality sequences and noise, including merging, filtering, and denoising, to generate high-quality effective data. Subsequent bioinformatic analyses, including taxonomic annotation and abundance profiling, were performed to characterize the microbial community composition. Library construction and sequencing were carried out by Majorbio Bio-Pharm Technology Co., Ltd. (Shanghai, China), and downstream data analyses were conducted on the Majorbio Cloud Platform (www.majorbio.com).

## Results

### Bioinformatic analysis

Using the Illumina MiSeq PE300 platform, the V3–V4 region of the 16S rRNA gene from gut microbiota of male and female plateau zokors were amplified and sequenced. After merging and quality filtering, a total of 279,150 high-quality sequences were obtained from 15 samples, including 148,880 sequences from females and 130,270 from males, with an average of 18,610 sequences per sample. In total, 2,963 amplicon sequence variants (ASVs) were identified, of which 2,099 ASVs were from female samples and 1,954 from male samples. The rank-abundance and rarefaction curves (Fig. S1) tended to approach saturation as sequencing depth increased, indicating that the sequencing effort was sufficient to capture the majority of microbial diversity, with only a limited number of additional taxa likely to be detected with further sequencing.

### Venn diagram analysis of gut microbiota in plateau zokors

A total of 1,090 ASVs were shared between female and male plateau zokors, while 1,009 ASVs were unique to females and 864 were unique to males. The gut microbial community in females exhibited greater richness compared with males. Specifically, ASVs from female and male samples accounted for 71.49% and 66.55% of the total microbial taxa, respectively (Fig. S2).

### Alpha diversity of gut microbiota in plateau zokors

Higher coverage values indicate a greater likelihood that all sequences within a sample have been captured. Both the female (FM) and male (M) groups showed coverage values of 99.4%, suggesting that only a small fraction of sequences remained undetected and that sequencing quality was high. In this study, the Shannon, Simpson, Ace, and Chao indices were applied to evaluate alpha diversity in the FM and M groups. The Ace and Chao indices are commonly used to assess community richness, with higher values indicating greater species numbers, whereas the Shannon and Simpson indices reflect community diversity, with Shannon positively and Simpson negatively correlated with diversity. As shown in Table 1, both richness and diversity were higher in females than in males; however, the differences were not statistically significant.

**Table 1:** Alpha diversity index table of FM group and F group (data are means ± SEM).

Sex	Shannon	Simpson	Ace	Chao	Coverage
FM	5.4165 ± 0.2721	0.0112 ± 0.0028	541.5385 ± 130.2516	542.7173 ± 131.3135	0.9994 ± 0.0005
M	5.1990 ± 0.4017	0.0195 ± 0.0194	536.4072 ± 71.5655	536.8930 ± 71.5207	0.9994 ± 0.0002
*P*	0.2533	0.1746	0.7723	0.7723	0.9074

### Beta diversity of gut microbiota in plateau zokors

**Figure 1:**
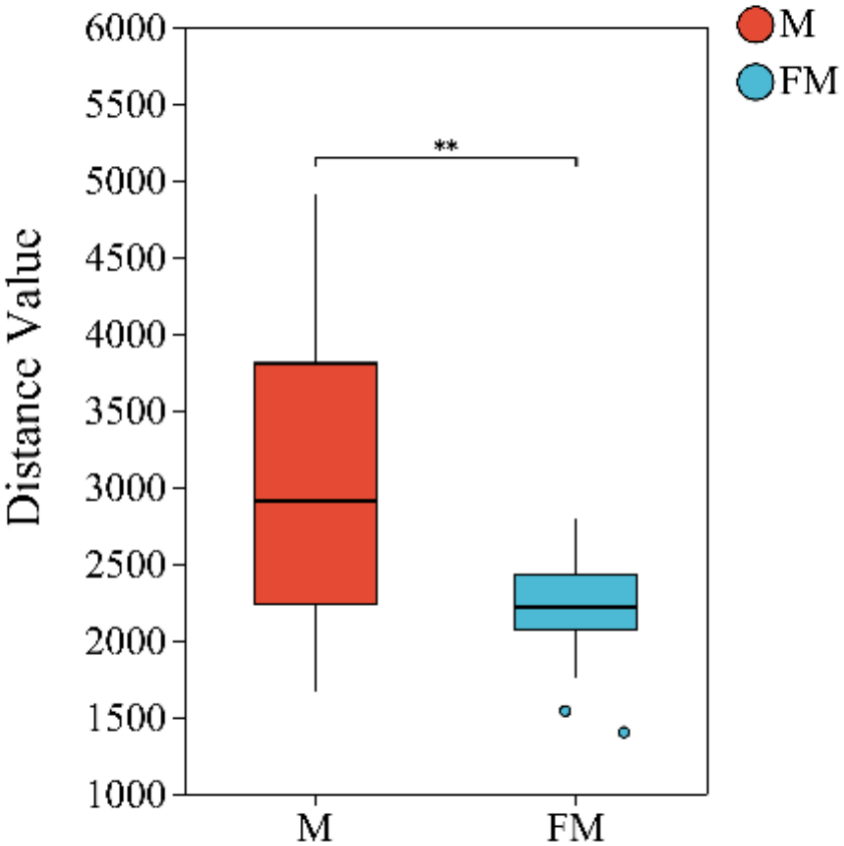
Beta diversity index difference analysis between groups. (0.001 < *p* ≤ 0.01 marked as **).

Beta diversity index difference analysis based on Euclidean distance was performed to compare microbial communities between the FM and M groups. The results revealed highly significant differences in gut microbial community structure between females and males (Female: 2,202.4 ± 341.3, Male: 2,999.4 ± 908.1, *p* = 0.003742 < 0.01). Moreover, intra-group variation was greater in males than in females (Fig. 1).

### Species composition of gut microbiota in plateau zokors

Excluding unclassified ASVs, a total of 10 phyla, 15 classes, 31 orders, 46 families, 101 genera, and 165 species were identified. A comparison of the number of microbial taxa at different taxonomic levels between the FM and M groups revealed that the species abundance of the gut microbiota in males was lower than in females (Table 2).

### Phylum-level species composition analysis

**Figure 2:**
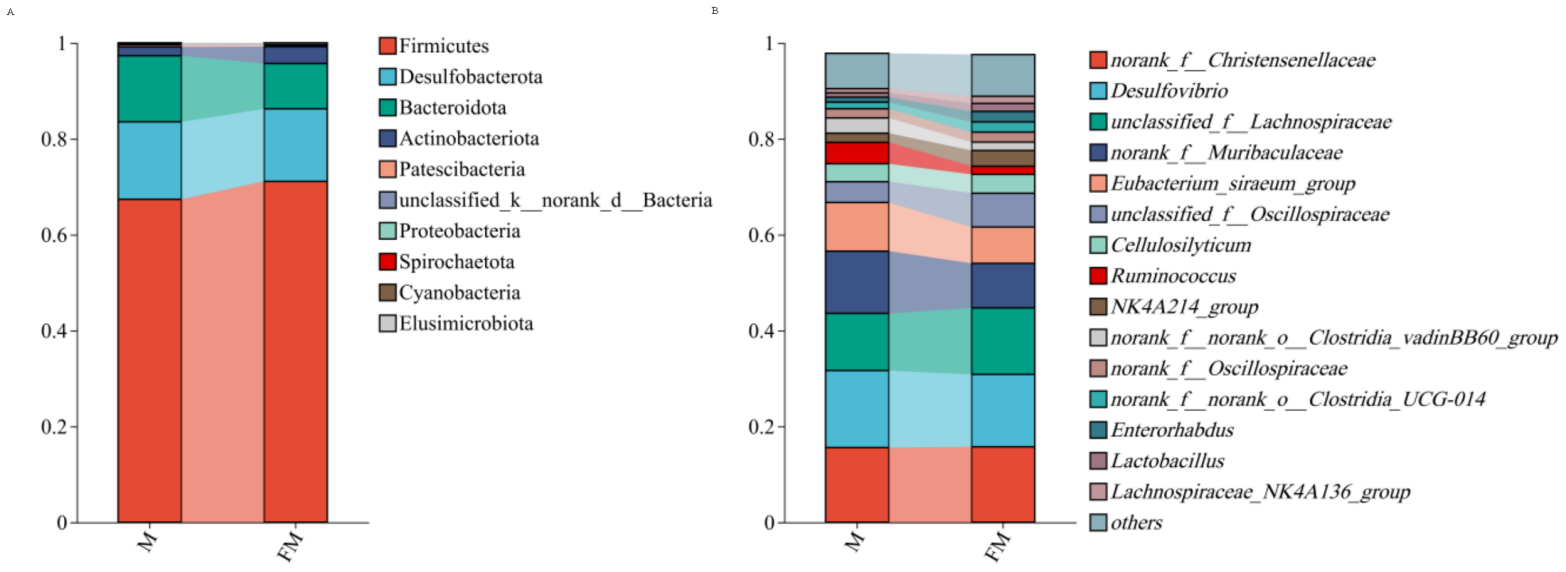
Relative abundance histograms at the phylum (A) and genus (B) level.

Figure 2A illustrates the relative abundance of gut microbiota at the phylum level in female and male plateau zokors. In female plateau zokors, the gut microbiota consisted of 10 phyla, with the dominant phyla being Firmicutes (71.10%), Desulfobacterota (15.14%), Bacteroidota (9.44%), and Actinobacteriota (3.53%). The predominant genera were Firmicutes, Desulfobacterota, and Bacteroidota. In male plateau zokors, the gut microbiota consisted of 9 phyla, with the major phyla being Firmicutes (67.37%), Desulfobacterota (16.17%), Bacteroidota (13.78%), and Actinobacteriota (1.85%). The dominant genera were also Firmicutes, Desulfobacterota, and Bacteroidota. Despite the presence of the same dominant phyla (Firmicutes, Desulfobacterota, and Bacteroidota) in both groups, their relative abundances differed.

### Genus-level species composition analysis

Figure 2B shows the relative abundance of gut microbiota at the genus level in female and male plateau zokors. In female plateau zokors, the gut microbiota consisted of 90 genera, with the predominant genera being norank_f__Christensenellaceae (15.74%), Desulfovibrio (15.12%), unclassified_f__Lachnospiraceae (13.86%), norank_f__Muribaculaceae (9.29%), Eubacterium_siraeum_group (7.58%), unclassified_f__Oscillospiraceae (7.05%), Cellulosilyticum (3.93%), NK4A214_group (3.27%), Enterorhabdus (2.18%), norank_f__norank_o__Clostridia_UCG-014 (2.13%), norank_f__Oscillospiraceae (2.09%), norank_f__norank_o__Clostridia_vadinBB60_group (1.75%), Ruminococcus (1.71%), Lactobacillus (1.66%), Lachnospiraceae_NK4A136_group (1.50%), Eubacterium_xylanophilum_group (1.27%), and Monoglobus (1.17%). The predominant genera were norank_f__Christensenellaceae, Desulfovibrio, and unclassified_f__Lachnospiraceae. In male plateau zokors, the gut microbiota consisted of 83 genera, with the dominant genera being Desulfovibrio (16.08%), norank_f__Christensenellaceae (15.58%), norank_f__Muribaculaceae (12.98%), unclassified_f__Lachnospiraceae (11.93%), Eubacterium_siraeum_group (10.15%), Ruminococcus (4.47%), unclassified_f__Oscillospiraceae (4.34%), Cellulosilyticum (3.74%), norank_f__norank_o__Clostridia_vadinBB60_group (3.18%), norank_f__Oscillospiraceae (1.92%), NK4A214_group (1.90%), norank_f__norank_o__Clostridia_UCG-014 (1.38%), Monoglobus (1.21%), and Enterorhabdus (1.03%). The predominant genera in male plateau zokors were Desulfovibrio, norank_f__Christensenellaceae, norank_f__Muribaculaceae, unclassified_f__Lachnospiraceae, and Eubacterium_siraeum_group. At the genus level, although both groups shared several dominant genera, their relative abundances were significantly different.

**Table 2:** Comparison of the number of different microbial classification units between FM group and F group.

Group	Phylum	Class	Order	Family	Genus	Species	ASV
FM	10	14	29	43	90	139	2,099
M	9	13	28	42	83	133	1,954
Total	10	15	31	46	101	165	2,963

### Functional prediction analysis of the gut microbiota in plateau zokors

The PICRUSt software is widely used for functional gene analysis and linking gut microbiota diversity to host metabolism. Functional analysis using the COG database (Fig. S3) revealed that the majority of genes in the gut microbiota of both female and male plateau zokors are related to metabolism, primarily involving energy production and conversion, as well as the transport and metabolism of inorganic ions, lipids, coenzymes, nucleotides, amino acids, carbohydrates, and amino acid metabolism. Additionally, genes associated with genetic and cellular functions were also identified, with major involvement in gene expression, transmission, regulation, repair, maintenance of genetic information, and the functionality of cellular biomembranes.

**Figure 3:**
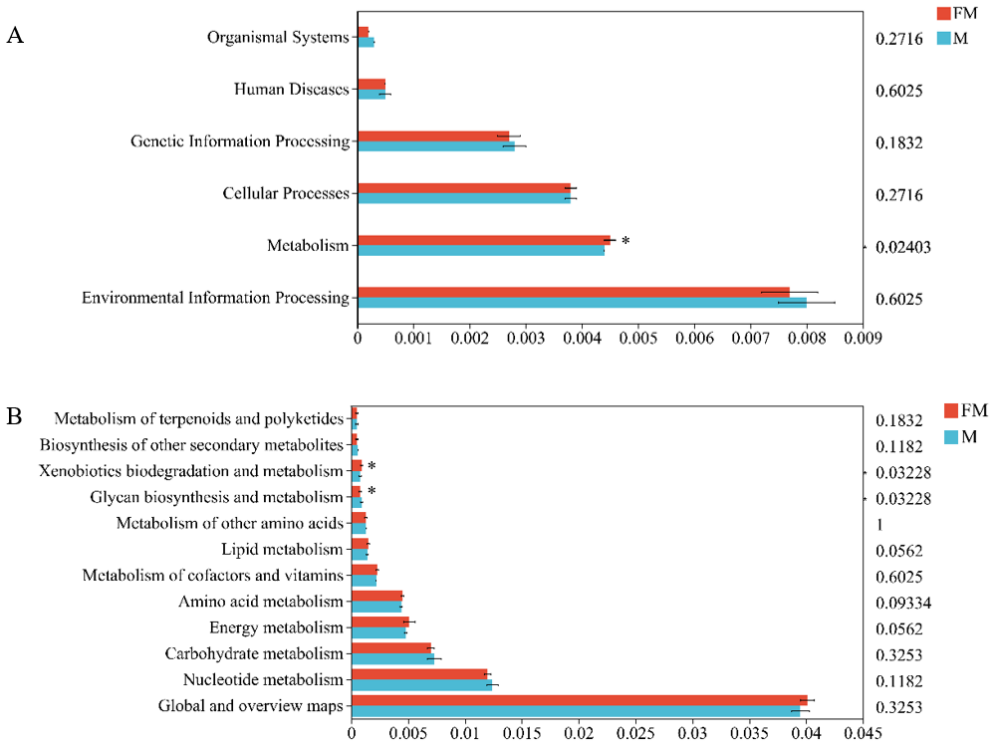
Differences in gut microbiota function between the two zokors at the level of KEGG level 1 (A) and level 2 (B). (0.01 < *p* ≤ 0.05 marked as *).

In the Kyoto Encyclopedia of Genes and Genomes (KEGG) pathway database, metabolic pathways at level 1 are classified into six major categories: organismal systems, environmental information processing, metabolism, cellular processes, genetic information processing, and human diseases. Using the Tax4Fun2 tool to align with the KEGG database, significant differences were observed between female and male plateau zokors in the metabolic functions within the six major biological functional categories (female: 0.0045 ± 0.0001, male: 0.0044 ± 0, *p* = 0.02403), particularly in the metabolism category (Fig. 3A).

A further abundance bar chart at the level 2 classification was plotted for each sample (Fig. 3B). At level 2 of the metabolic pathways, 12 functional categories were identified, with the highest abundance found in “Global and overview maps”, followed by “Nucleotide metabolism”, “Carbohydrate metabolism”, “Energy metabolism”, and “Amino acid metabolism”, among others. Differential analysis of metabolic pathways revealed significant functional differences between the two types of zokors. Specifically, “Glycan biosynthesis and metabolism” was significantly enriched in male plateau zokors (Female: 0.0008 ± 0.0001, Male: 0.0009 ± 0.001, *p* = 0.03228), while “Xenobiotics biodegradation and metabolism” was significantly enriched in female plateau zokors (female: 0.0008 ± 0.0001, male: 0.0009 ± 0.001, *p* = 0.03228).

### Community Assembly Process of Gut Microbiota

**Figure 4:**
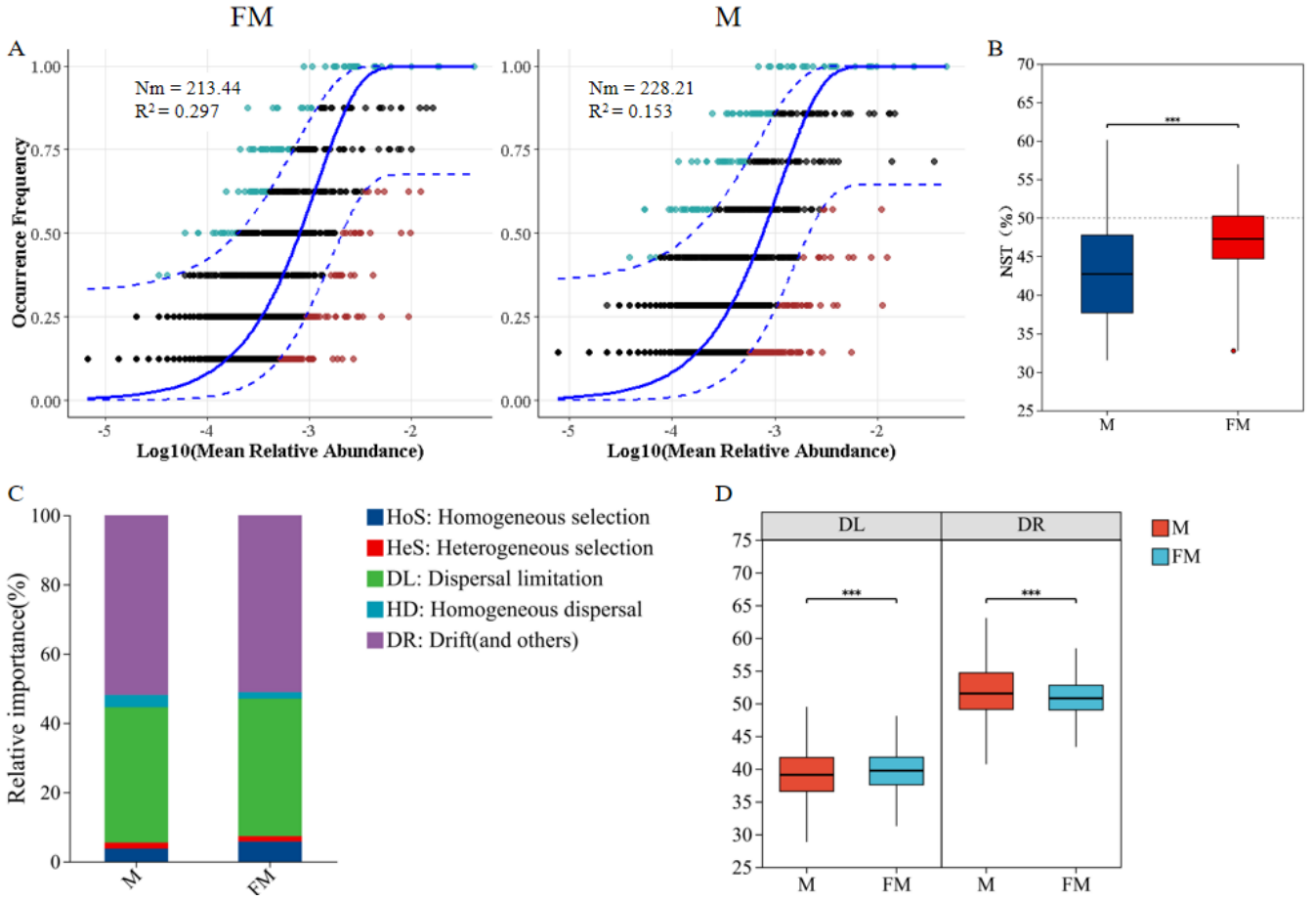
Neutral community model (NCM) of male and female plateau zokor gut microbiota (A); Standardized random rate (NST) of male and female gut microbiota (B); Zero model analysis of gut microbiota (C); Ecological process comparison of gut microbiota (D). (*p* ≤ 0.001 marked as ***) Note: The neutral community model best fit (blue solid line) and its 95% prediction interval (blue dashed line); ASVs within (black) and outside (colored) the prediction interval; *R*^2^ (goodness-of-fit) and Nm (community size × migration rate) are shown.

The neutral community model (NCM) was used to quantify the importance of neutral processes in the assembly of gut microbiota communities. The results showed that the Nm value for male plateau zokors were higher than that for females (Fig. 4A), indicating a more extensive species dispersal of the gut microbiota in male individuals. The goodness of fit for both male and female gut microbiota communities was low (R^2^ < 0.3), suggesting that random processes contribute minimally to community assembly. Both deterministic and stochastic processes are involved in the formation of the gut microbiota communities in plateau zokors. The goodness of fit for males (*R*^2^ = 0.153) was lower than for females (*R*^2^ = 0.297), indicating that the gut microbiota community structure in females more closely align with the neutral theory hypothesis. Stochastic processes had a greater impact on the diversity of female gut microbiota, while deterministic processes had a greater influence on the assembly of the male gut microbiota.

The standardized neutrality score (NST) was used to assess the relative importance of random and deterministic processes in the gut microbiota of male and female plateau zokors (Zhang & Liu, 2022). The results indicated that both male and female plateau zokors had NST values below the 50% threshold, suggesting that deterministic processes dominate in both sexes (Fig. 4B). Notably, the NST value for males was significantly lower than that for females (female: 0.4706 ± 0.0441, male: 0.4319 ± 0.0679, *p* < 0.001), indicating that the gut microbiota community in males undergoe a more deterministic assembly process compared to females.

Further, the zero-model analysis based on phylogenetic bins (iCAMP) classified random and deterministic processes into different ecological processes to estimate the relative importance of each (Ning et al., 2019). The results revealed that the assembly mechanisms of the gut microbiota in both male and female plateau zokors were predominantly driven by drift (DR) and diffusion limitation (DL), followed by homogeneous selection (HoS), homogeneous diffusion (HD), and heterogeneous selection (HeS) (Fig. 4C). Significant differences were observed in the ecological processes between the sexes (Fig. 4D). Specifically, diffusion limitation (female: 39.72 ± 3.16, male: 43.19 ± 6.79) and homogeneous selection (female: 5.54 ± 1.76, male: 3.56 ± 0.86) were significantly higher in females than in males (*p* < 0.001), while drift (female: 50.85 ± 2.8, male: 51.79 ± 4.42), homogeneous diffusion (female: 1.84 ± 0.88, male: 3.39 ± 1.63), and heterogeneous selection (female: 1.52 ± 0.4, male: 1.71 ± 0.5) were significantly higher in males than in females (*p* < 0.001).

## Discussion

### Significant differences in gut microbial diversity between male and female plateau zokors

Sex differences in gut microbiota diversity have been suggested to result from the influence of sex hormones and other genetic factors (Mendelsohn & Karas, 2005; Bolnick et al., 2014). In this study, differences in the gut microbiota between male and female Plateau zokors were analyzed using Venn diagrams, alpha diversity indices, and beta diversity analysis. The results indicated significant differences in the gut microbial communities between male and female plateau pikas. The alpha diversity analysis revealed no significant differences in species abundance or diversity between the sexes, which is consistent with findings from studies on the black-tailed gerbil (Liu, 2022) and Yunnan golden monkeys (Yu, 2020). However, the beta diversity analysis demonstrated highly significant differences in the gut microbial structure between the male and female groups, with greater variation observed within the male group compared to the female group. This pronounced difference could be attributed to factors such as differing genotypes, hormonal fluctuations, and dietary variations between male and female Plateau zokors (Kovacs et al., 2011; Costello et al., 2012; Freire et al., 2011). Additionally, as the samples in this study were collected during winter, a period when male Plateau zokors engage in more activity (*e.g.*, foraging, digging, and movement) than females (Zhou & Dou, 1990), the males require larger amounts of food, resulting in differences in resource acquisition and, consequently, greater variation in gut microbiota between male samples. Conversely, female samples exhibited higher similarity in microbiota composition, a finding consistent with the study by Yang et al. (2018) on the structural differences in the gut microbiota between male and female Gansu zokor (*Eospalax cansus*).

### Sex-dependent variations in gut microbial composition of plateau zokors

The dominant gut microbiota of male and female Plateau zokors overlapped at both the phylum and genus levels, although the abundance of these dominant groups differed. At the phylum level, the dominant gut microbiota in both male and female Plateau zokors consisted of Firmicutes, Desulfobacterota, and Bacteroidota. Firmicutes, a predominant phylum commonly found in vertebrates, plays a crucial role in absorbing nutrients from food. Many Firmicutes species are involved in digestion and metabolism, particularly in the breakdown of complex carbohydrates, which generate energy for the host (Flint et al., 2012). Bacteroidota is primarily involved in metabolic processes such as carbohydrate and sugar metabolism, enhancing the host’s ability to absorb and utilize nutrients (Hooper, 2004; Hooper et al., 2001), thus maintaining the balance of the gut microbiota. Firmicutes and Bacteroidota are primarily involved in fiber degradation in the gut, aiding in the digestion of plant-based foods. For instance, herbivores such as the Gansu zokor, plateau pikas, horses, and donkeys all exhibit a high proportion of Firmicutes and Bacteroidota in their gastrointestinal tract (Ren et al., 2020; Tan, Li & Qu, 2019; Costa et al., 2017; Liu et al., 2014), which aligns with the dietary characteristics of plateau pikas. In this study, the ratio of Firmicutes to Bacteroidota was higher in female Plateau zokors compared to males. Previous studies have indicated that obese individuals exhibit a significantly higher Firmicutes-to-Bacteroidota ratio compared to individuals with normal body weight (Patrone et al., 2016), suggesting that female Plateau zokors may need to store more fat to meet the energy demands during reproduction, particularly in the limited food supply during winter. At the genus level, the common dominant microbiota in both male and female Plateau zokors included Desulfovibrio, norank_f__Christensenellaceae, and unclassified_f__Lachnospiraceae. Desulfovibrio is a common pathogenic genus that can reduce sulfate to sulfide, which is toxic to intestinal epithelial cells, leading to abnormal proliferation and metabolism of these cells (Gao et al., 2017). Additionally, Desulfovibrio disrupts the intestinal barrier function by inhibiting butyrate oxidation (Pitcher & Cummings, 1996). Both norank_f__Christensenellaceae and unclassified_f__Lachnospiraceae are butyrate-producing bacteria (Shang et al., 2019). The presence of these bacteria may serve as a compensatory mechanism to counteract the inhibitory effect of Desulfovibrio, maintaining the homeostasis of the gastrointestinal environment in plateau pikas.

### Marked differences in metabolic pathways of gut microbiota in plateau zokors

PICRUSt was utilized to investigate the relationship between gut microbiota, the host, and the environment (Chen et al., 2021). The COG functional classification results indicated that the majority of genes in the gut microbiota of Plateau zokors are associated with metabolism. Genes related to amino acid and carbohydrate metabolism, as well as energy production and conversion, were found to be abundant. This is consistent with the involvement of the dominant gut microbiota (Firmicutes and Bacteroidota) in host glucose and carbohydrate metabolism (Flint et al., 2012; Hooper, 2004; Hooper et al., 2001). A study by Ma et al. (2019a) and Ma et al. (2019b) found that under hypoxic conditions in the plateau, rat gut microbiota may contribute to host energy metabolism by enhancing gluconeogenesis and amino acid metabolism. Similarly, Plateau zokors may adapt to the high-altitude hypoxic environment by strengthening amino acid metabolism. Some genes were related to gene repair and cellular defense mechanisms, suggesting that either the gut microbiota or the host itself might experience genetic damage, possibly due to long-term exposure to high-altitude hypoxia (An et al., 2024). At the KEGG level 1, significant differences were observed between male and female Plateau zokors in metabolic functions. At the level 2 pathway, glycan biosynthesis and metabolism were significantly enriched in males, indicating that male Plateau zokors have a stronger capacity for carbohydrate metabolism compared to females. This could be attributed to the higher activity levels and greater energy requirements of males, which lead them to consume more high-carbohydrate plant roots and tubers (Zhou & Dou, 1990; Zhang et al., 2020). Xenobiotic biodegradation and metabolism were significantly enriched in females, suggesting that females have a greater capacity for metabolizing toxins or pharmaceuticals compared to males, possibly due to hormonal differences. It is important to note that the functional profiles discussed here are based on predictions from 16S rRNA gene data using PICRUSt, rather than direct measurements of functional genes. Therefore, these findings represent inferred functional potentials rather than confirmed metabolic activities. Future studies employing shotgun metagenomic sequencing would be valuable to validate these predicted functional differences.

### Distinct assembly processes of gut microbiota between male and female plateau zokors

Microbial community assembly processes are key mechanisms that shape microbial diversity, function, and distribution. They are major determinants of gut microbiota composition (Chen et al., 2019). Most studies suggest that both deterministic and stochastic processes interact in shaping microbial communities, rather than operating in isolation (Yu, Li & Li, 2022; Nemergut et al., 2013). In this study, NCM analysis indicated that both deterministic and stochastic processes play roles in the assembly of the gut microbiota in plateau pikas. The gut microbiota of female Plateau zokors conformed more closely to the neutral theory hypothesis, with stochastic processes having a greater impact on their gut microbiota diversity, whereas deterministic processes were more influential in the assembly of the male microbiota. Additionally, species dispersal between males was broader than in females, which could be attributed to the larger activity range of male plateau pikas, allowing for greater microbial transmission between individuals. NST analysis showed that deterministic processes dominate the assembly of both male and female plateau pika microbiota, with males exhibiting a more deterministic assembly process compared to females, further supporting the results of the NCM analysis. iCAMP analysis revealed that the assembly of both male and female plateau pika microbiota were influenced by stochastic processes, such as drift and dispersal limitation, as well as deterministic factors, such as selection, with significant differences between the sexes. Drift and dispersal limitation were found to be the primary assembly processes of the plateau pika gut microbiota, which is consistent with the findings of Zou et al. (2024) in herbivores’ bacterial community assembly. The drift process in males was significantly higher than in females, which is in line with studies suggesting that communities with lower biomass and richness are more prone to drift or founder effects (Vellend et al., 2014; Evans, Martiny & Allison, 2017). In this study, males exhibited lower diversity and richness in their gut microbiota compared to females, making them more susceptible to drift. The dispersal limitation process was significantly higher in female plateau pikas, indicating that, compared to females, the microbial species in males’ gut microbiota are more likely to disperse between individuals, further validating the results obtained from the NCM analysis.

## Conclusions

In conclusion, although species richness and diversity did not differ significantly between sexes, the gut microbial community structure, dominant taxa, and metabolic functions showed pronounced sex-specific differences in plateau zokors. Males exhibited higher carbohydrate metabolism, while females showed stronger xenobiotic metabolism, with microbiota assembly more deterministic in males. These results demonstrate that sex influences gut microbiota composition, function, and assembly, contributing to host adaptation to high-altitude environments. Future metagenomic and metabolomic studies are required to elucidate the functional relevance of these sex differences.

## Supplemental Information

10.7717/peerj.20646/supp-1 Supplemental Information 1 The rank-abundance curve (A) and the rarefaction curve (B) of intestinal microbes in plateau zokor

10.7717/peerj.20646/supp-2 Supplemental Information 2 Venn diagram of intestinal microbial community composition of plateau zokor

10.7717/peerj.20646/supp-3 Supplemental Information 3 COG function prediction

## Additional Information and Declarations

### Data Availability

The following information was supplied regarding data availability: The data is available at NGDC: PRJCA046519, CRA030652.

## References

[ref-1] An X, Mao L, Wang Y, Xu QQ, Liu X, Zhang SZ, Qiao ZL, Li BW, Li F, Kuang ZR, Wan N, Liang XL, Duan QJ, Feng ZL, Yang XJ, Liu SY, Nevo E, Liu JQ, Storz JF, Li KX (2024). Genomic structural variation is associated with hypoxia adaptation in high-altitude zokors. Nature Ecology and Evolution.

[ref-2] Bolnick DI, Snowberg LK, Hirsch PE, Lauber CL, Org E, Parks B, Lusis AJ, Knight R, Caporaso JG, Stanback R (2014). Individual diet has sex-dependent effects on vertebrate gut microbiota. Nature Communications.

[ref-3] Chen XN, Feng T, Hou X, An XL, Wang J, Chang G (2021). A preliminary analysis of the intestinal microbial community and function prediction of female Myospalax rothschildi. Chinese Journal of Wildlife.

[ref-4] Chen WD, Ren KX, Isabwe A, Chen HH, Liu M, Yang J (2019). Stochastic processes shape microeukaryotic community assembly in a subtropical river across wet and dry seasons. Microbiome.

[ref-5] Chu B, Bao DEH, Ye GH, Hua R, Zhou R, Zhang FY, Tang ZS, Hao YY, Hua LM (2023). Habitat suitability of the plateau zokor (*Eospalax baileyi*) in the eastern Tibetan Plateau. Chinese Journal of Grassland.

[ref-6] Costa MC, Arroyo LG, Allen-Vercoe E, Stampfli HR, Kim PT, Sturgeon A, Weese JS (2017). Comparison of the fecal microbiota of healthy horses and horses with colitis by high-throughput sequencing of the V3-V5 region of the 16S rRNA gene. PLOS ONE.

[ref-7] Costello EK, Stagaman K, Dethlefsen L, Bohannan BJM, Relman DA (2012). The application of ecological theory toward an understanding of the human microbiome. Science.

[ref-8] Derrien M, Alvarez AS, Vos WMD (2019). The gut microbiota in the first decade of life. Trends in Microbiology.

[ref-9] Ding Y, Wu Q, Hu YB, Wang X, Nie YG, Wu XG, Wei FE (2017). Research progress and prospects of intestinal microbiomes in wild mammals. Acta Theriologica Sinica.

[ref-10] Evans S, Martiny JBH, Allison SD (2017). Effects of dispersal and selection on stochastic assembly in microbial communities. ISME.

[ref-11] Ewen C (2017). ‘Young poo’ makes aged fish live longer. Nature.

[ref-12] Flint HJ, Scott KP, Louis P, Duncan SH (2012). The role of the gut microbiota in nutrition and health. Nature Reviews Gastroenterology & Hepatology.

[ref-13] Freire AC, Basit AW, Choudhary R, Piong CW, Merchant HA (2011). Does sex matter? The influence of gender on gastrointestinal physiology and drug delivery. International Journal of Pharmaceutics.

[ref-14] Gao XJ, Li T, Wei B, Yan ZX, Yan R (2017). Regulatory mechanisms of gut microbiota on intestinal CYP3A and P-glycoprotein in rats with dextran sulfate sodiuminduced colitis. Acta Pharmacolsin.

[ref-15] Hooper LV (2004). Bacterial contributions to mammalian gut development. Trends in Microbiology.

[ref-16] Hooper LV, Wong MH, Thelin A, Thelin A, Hansson L, Falk PG, Gordon JI (2001). Molecular analysis of commensal host-microbial relationships in the intestine. Science.

[ref-17] Kovacs A, Ben-Jacob N, Tayem H, Halperin E, Iraqi FA, Gophna U (2011). Genotype is a stronger determinant than sex of the mouse gut microbiota. Microbial Ecology.

[ref-18] La MC, Wang Y, He ZQ, Wang K, Geng YYP, Niu PL, Wang W, Liu HJ, Zhou S (2025). Composition and function of gut microbiota in plateau zokors and its adaptation to the alpine grassland environment in Western Sichuan. Journal of Grassland Science.

[ref-19] Li Z, Wright ADG, Liu H, Bao K, Zhang TT, Wang KY, Cui XZ, Yang FH, Zhang ZG, Li GY (2015). Bacterial community composition and fermentation patterns in the rumen of sika deer (Cervus Nippon) fed three different diets. Microbial Ecology.

[ref-20] Liu H (2022). Seasonal changes and gender differences of gut microbiota in striped hamsters under semi-natural conditions.

[ref-21] Liu XF, Fan HL, Ding XB, Ding XB, Hong ZS, Nei YW, Liu ZW, Li GP, Guo H (2014). Analysis of the gut microbiota by high-throughput sequencing of the V5-V6 regions of the 16S rRNA gene in donkey. Current Microbiology.

[ref-22] Liu S, Imad S, Hussain S, Xiao SQ, Yu XW, Cao H (2024). Sex, health status, and habitat alter the community composition and assembly processes of symbiotic bacteria in captive frogs. BMC Microbiology.

[ref-23] Ma Y, Ma S, Shang CX, Ge RL (2019a). Effects of hypoxic exposure on rats’ gut microbiota. Microbiology China.

[ref-24] Ma SJ, Zhou JW, Wang FC, Niu YJ, Chu B, Zhou YS, Ji CP, Wang T, Hua LM (2019b). Effect of soil erosion of plateau Zokor New Mound in Alpine Meadow. Journal of Soil and Water Conservation.

[ref-25] Mendelsohn ME, Karas RH (2005). Molecular and cellular basis of cardiovascular gender differences. Science.

[ref-26] Nemergut DR, Schmidt SK, Fukami T, O’Neill SP, Bilinski TM, Stanish LF, Knelman JE, Darcy JL, Lynch RC, Wickey P, Ferrenberg S (2013). Patterns and processes of microbial community assembly. Microbiology and Molecular Biology Reviews.

[ref-27] Nevo E (2007). Mosaic evolution of subterranean mammals: tinkering, regression, progression, and global convergence. Subterranean Rodents.

[ref-28] Ning D, Deng Y, Tiedje J, Zhou JZ (2019). A general framework for quantitatively assessing ecological stochasticity. Proceedings of the National Academy of Sciences of the United States of America.

[ref-29] Norris RW, Zhou K, Zhou C, Yang G, Kilpatrick CW, Honeycutt RL (2004). The phylogenetic position of the zokors (Myospalacinae) and comments on the families of muroids (Rodentia). Molecular Phylogenetics and Evolution.

[ref-30] Org E, Mehrabian M, Parks BW, Shipkova P, Liu XQ, Drake TA, Lusis AJ (2016). Sex differences and hormonal effects on gut microbiota composition in mice. Gut Microbes.

[ref-31] Patrone V, Vajana E, Minuti A, Callegari ML, Federico A, Loguercio C, Dallio M, Tolone S, Docimo L, Morelli L (2016). Postoperative changes in fecal bacterial communities and fermentation products in obese patients undergoing bilio-intestinal bypass. Frontiers in Microbiology.

[ref-32] Pitcher MC, Cummings JH (1996). Hydrogen sulphide: a bacterial toxin in ulcerative colitis?. Gut.

[ref-33] Raymann K, Moran NA (2018). The role of the gut microbiome in health and disease of adult honeybee workers. Current Opinion in Insect Science.

[ref-34] Ren SE, Nan XN, Xu M, Xu M, Zhou Y, Lang NN, Shi JN, Han CX (2020). Comparison of intestinal bacterial diversity of Gansu zokor under wild and artificial feeding conditions. Acta Microbiologica Sinca.

[ref-35] Ren S, Yang J, Aba X, Hu Y, Zhao YF, Pang SS, Han CX, Zhang LZ, Nan XN (2025). Grazing is associated with dietary diversity and gastrointestinal microbiota in Subterranean rodents. Ecology and Evolution.

[ref-36] Shang QH, Liu HS, Liu SJ, He TF, Piao XS (2019). Effects of dietary fiber sources during late gestation and lactation on sow performance, milk quality, and intestinal health in piglets. Journal of Animal Science.

[ref-37] Shi BN, Zhao Y, Lin ZH, Tian JX, Sun J, Ma YW, Liu ZS, Teng LW (2023). Gender differences in intestinal microbiota composition of Captive Sika Deer. Chinese Journal of Wildlife.

[ref-38] Tan YC (2024). Multi-omics response characteristics of plateau zokor (*Eospalax baileyi*) to Stellera chamaejasme complex.

[ref-39] Tan CT, Li H, Qu JP (2019). Comparison of gut microbial diversity between wild and laboratory-reared plateau pikas. Pratacultural Science.

[ref-40] Tetel MJ, De Vries GJ, Melcangi RC, Panzica G, O’Mahony SM (2018). Steroids, stress and the gut microbiome-brain axis. Journal of Neuroendocrinology.

[ref-41] Tremaroli V, Bäckhed F (2012). Functional interactions between the gut microbiota and host metabolism. Nature.

[ref-42] Vellend M, Srivastava DS, Anderson KM, Brown CD, Jankowski JE, Kleynhans EJ, Kraft NJB, Letaw AD, Macdonald AAM, Maclean JE, Myers-Smith IH, Norris AR, Xue XX (2014). Assessing the relative importance of neutral stochasticity in ecological communities. Oikos.

[ref-43] Wei WR (2018). Study on the population dynamics of plateau pika and plateau zokor and their relationship with vegetation.

[ref-44] Xu XM, Zhang ZB (2021). Sex- and age-specific variation of gut microbiota in Brandt’s voles. PeerJ.

[ref-45] Yang J, Nan XN, Zou Y, Zhang FR, Shi JN, Han CX (2018). Effects of the three factors on intestinal bacterial diversity of *Eospalax cansus* in the region of Liupan Mountains. Acta Microbiologica Sinca.

[ref-46] Yu XL (2020). Differences in intestinal microbial community structure between male and female Yunnan snub-nosed monkeys in different seasons.

[ref-47] Yu Q, Li G, Li H (2022). Two community types occur in gut microbiota of large-sample wild plateau pikas (Ochotona curzoniae). Integrative Zoology.

[ref-48] Zhang YM, Liu JK (2002). Effect of plateau zokors on vegetation characteristics and productivity of alpine meadow. Acta Theriologica Sinica.

[ref-49] Zhang FY, Zhou JW, Zhou FF, Zhou R, Hua XZ, Hua LM (2020). The change of home range of plateau zokor during courtship period and its relationship with body mass. Grassland and Turf.

[ref-50] Zhou WY, Dou FM (1990). Studies on activity and home range of plateau zokor. Acta Theriologica Sinica.

[ref-51] Zhu D, Lu L, Zhang Z, Qi DW, Zhang MC, O’Connor P, Wei FW, Zhu YG (2021). Insights into the roles of fungi and protists in the giant panda gut microbiome and antibiotic resistome. Environment International.

[ref-52] Zou H, Li Q, Liu J, Wang XT, Gao Q, Yang YF, Zhao XQ (2024). Fecal microbiota reveal adaptation of herbivores to the extreme environment of the Qinghai-Tibet Plateau. Grassland Research.

