# Supplementary figures and images for: Sex differences in gut microbiota composition, function, and assembly in the plateau zokor (Eospalax baileyi)
Source: PeerJ. 2026 Jan 26;14:e20646. doi: 10.7717/peerj.20646 (PMC12854129; doi:10.7717/peerj.20646)

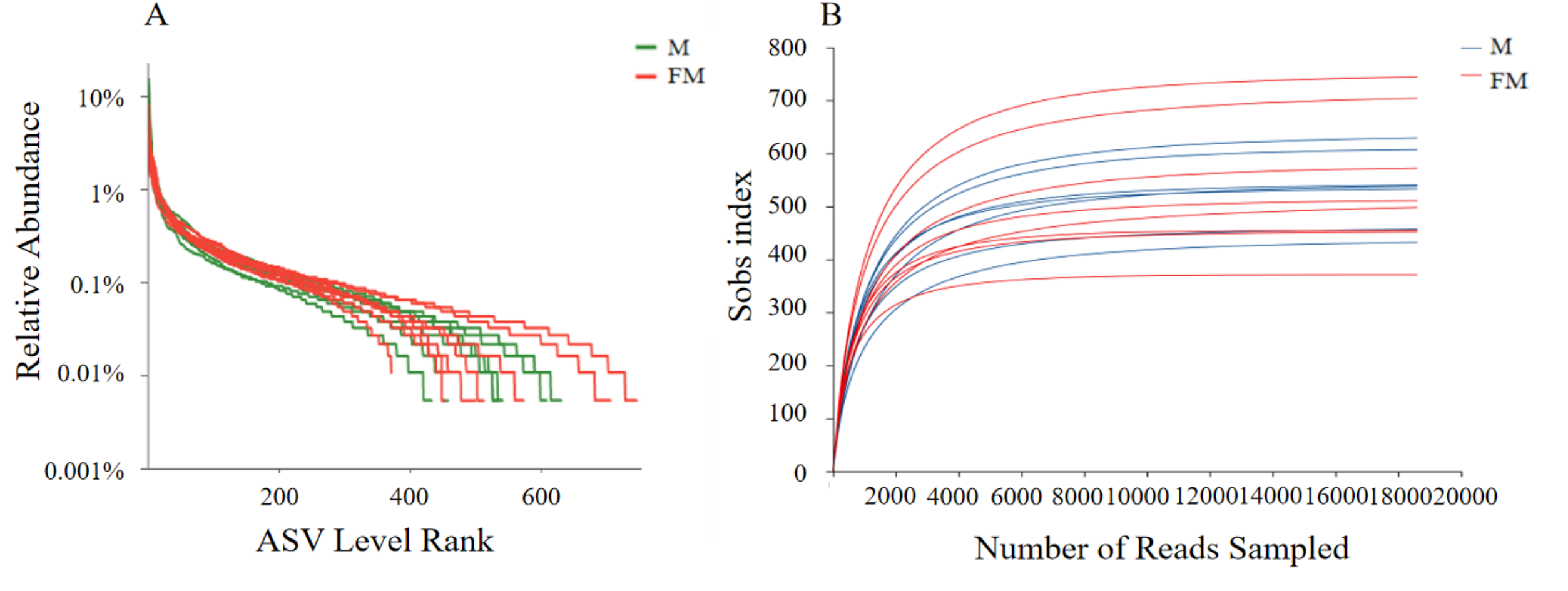

Supplement: Supplemental Information 1 [file peerj-14-20646-s001.png]

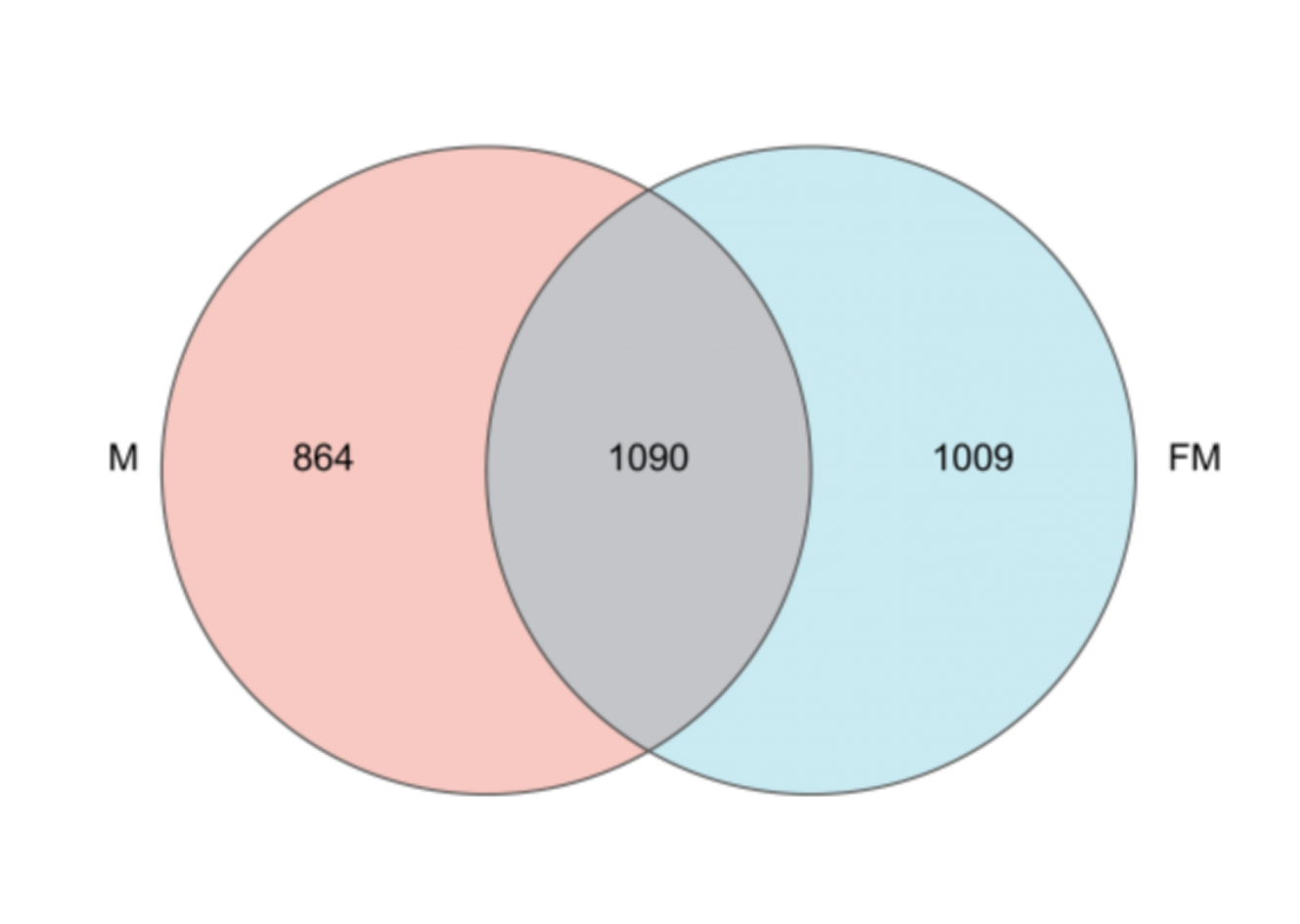

Supplement: Supplemental Information 2 [file peerj-14-20646-s002.png]

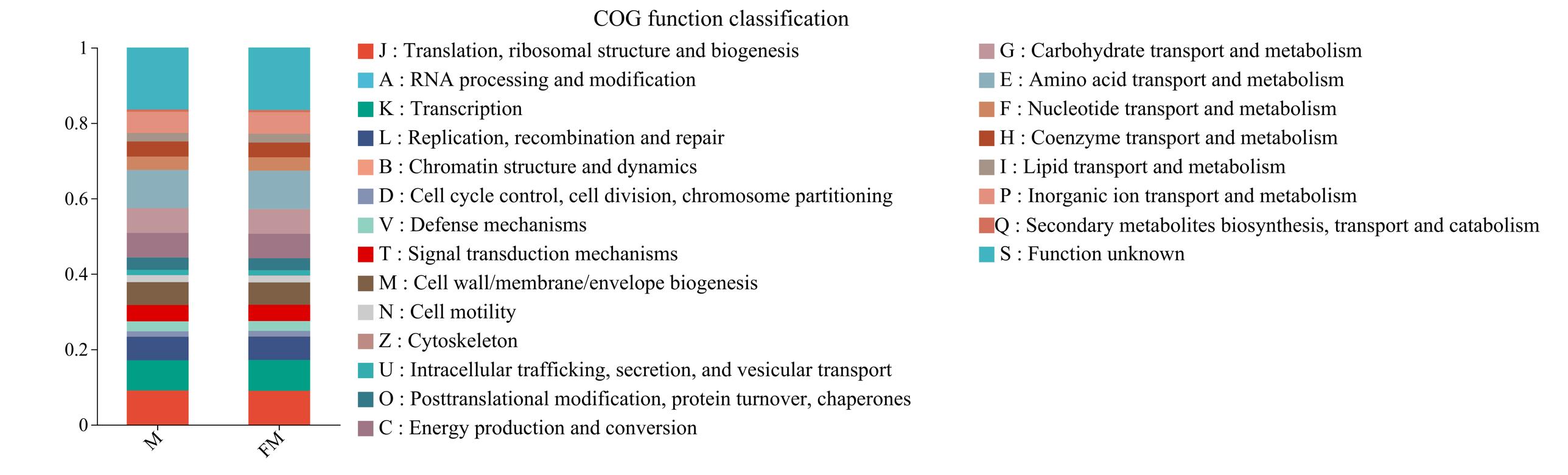

Supplement: Supplemental Information 3 [file peerj-14-20646-s003.png]
